# Low regulatory T-cells frequency is associated with graft rejection after small bowel transplantation: Clinical and experimental evidence

**DOI:** 10.1371/journal.pone.0307534

**Published:** 2025-01-24

**Authors:** Rodrigo Papa-Gobbi, Pablo Stringa, Maria Virginia Gentilini, Ivana Ivanoff, Mariana Machuca, Nidia Monserrat Arreola, Javier Serradilla, Karla Estefanía-Fernández, Paloma Talayero, María Velayos, Elena Sánchez—Zapardiel, Gabriel Gondolesi, Ane Andrés-Moreno, Martin Rumbo, Francisco Hernández-Oliveros

**Affiliations:** 1 Institute for Immunological and Pathophysiological Studies (IIFP), School of Exact Sciences, National University of La Plata, National Council of Scientific and Technical Research (CONICET), La Plata, Argentina; 2 Intestinal Failure, Rehabilitation and Transplant Unit, University Hospital Foundation Favaloro; Institute of Translational Medicine, Transplantation and Bioengineering (ImeTTyB), University Favaloro-CONICET, Buenos Aires, Argentina; 3 Special Pathology Laboratory, Faculty of Veterinary Sciences, National University of La Plata, La Plata, Buenos Aires, Argentina; 4 Transplant Group, La Paz University Hospital Health Research Institute (IdiPAZ), Madrid, Spain; 5 Department of Pediatric Surgery, La Paz University Hospital, Madrid, Spain; 6 Immunology Department, 12 de Octubre University Hospital, Madrid, Spain; 7 Immunology Department, La Paz University Hospital, Madrid, Spain; Medical University of Gdansk, POLAND

## Abstract

**Background:**

Intestinal transplantation (ITx) represents the only curative option for patients with irreversible intestinal failure. Nevertheless, its rejection rate surpasses that of other solid organ transplants due to the heightened immunological load of the gut. Regulatory T-cells (Tregs) are key players in the induction and maintenance of peripheral tolerance, suggesting their potential involvement in modulating host *vs*. graft responses after ITx. Thus, we investigated the association of Tregs with allograft outcomes in pediatric patients and in an experimental model of small bowel transplantation.

**Methods:**

Treg frequency in human samples was analyzed by Flow cytometry (CD4^+^CD25^high^CD127^-^, blood samples) and immunohistochemistry (FoxP3, graft samples). Experimental allogenic-heterotopic small bowel transplantation was performed in rats and animals divided into 3 groups: non-immunosuppressant treatment, rapamycin (2 mg/kg), and tacrolimus (0.6 mg/kg) treatment. Acute cellular rejection (ACR) was diagnosed based on clinical and histological findings, graft gene expression of pro- and anti-inflammatory mediators assessed by RT-qPCR, serum IL-6 and IL-10 levels by Luminex, and Treg frequency analyzed by flow cytometry (CD4^+^CD25^high^FoxP3^+^).

**Results:**

Blood samples from patients undergoing ACR exhibited a significant reduction in the Treg number compared to those with normo-functional grafts. Similarly, a diminished number of FoxP3^+^ cells was observed in mucosa samples with ACR. In the experimental model, rapamycin-treated animals displayed clinical and histological findings resembling those not receiving immunosuppression treatment. Notably, ACR correlated with a high CD8/CD4 ratio, loss of T-cell chimerism, mRNA upregulation of pro-inflammatory genes and diminished graft Treg frequency. In contrast, tacrolimus treatment prevented ACR and facilitate blood and graft Treg expansion. Remarkably, recipients who achieved Treg expansion within the graft remained free of ACR even after discontinuation of the immunosuppressant treatment and this phenomenon was associated with increased levels of serum IL-10.

**Conclusion:**

Our clinical and experimental findings underscore the association between Treg frequency and graft rejection after ITx, advocating for strategies that promote their expansion within the gut mucosa to enhance long-term outcomes.

## Introduction

Intestinal transplantation (ITx) represents the only curative treatment for patients with organ incapacity to fulfill the basic absorption, nutritional, and growth requirements; including: short gut syndrome, motility disorders, malabsorption, and tumors, among others [1, 2]. However, the immunological complexity of the intestine leads to a higher incidence of rejection compare to other solid organ transplants; 50–75% of patients experience at least one episode of acute cellular rejection (ACR) [2, 3], with significant graft loss rates and a necessity for re-transplantation at 1, 5, and 10 years of 29%, 50%, and 59%, respectively [1, 2]. Moreover, the life-long complications arising from the high doses of immunosuppressive drugs required to prevent allogenic responses, including infections, malignancies, renal failure, cardiovascular disease, and others, substantially diminish patient well-being [4] (10-year survival of only 43%) [1, 2]. Therefore, strategies aimed at modulating the host *vs*. graft response without eliciting pharmacological side effects will significantly enhance ITx recipient outcomes and improve cost efficiency within the healthcare system.

CD4^+^ regulatory T-cells (Tregs) represent a specialized subset of lymphocyte capable of suppressing pro-inflammatory responses through diverse array of pathways [5–7], thereby assuming a central role in tolerance induction and maintenance [8, 9]. Operational tolerance have been linked to increased Treg frequency in patients who undergone kidney and liver transplants, contributing to enhanced long-term allograft survival [10, 11]; and their potential as cell-based therapeutic agents holds considerable promise for future medical interventions [12, 13]. In the context of ITx, the implementation of the “Leuven Immunomodulatory Protocol” has yielded significant improvements in the 5-year patient/graft survival, alongside reduction in both early and late ACR occurrence [14]. This favorable outcome has been associated with the expansion of peripheral blood Tregs, mirroring findings in tolerant kidney recipients. On the contrary, diminished Treg frequency heightens the risk of graft rejection [15, 16], which is important because chronic immunosuppressive therapies dampen both tissue-destructive and tissue-protective T-cells [17].

As the understanding of Tregs biology in the context of ITx remains limited, we investigated their frequency in a pediatric cohort of ITx patients, as well as in an experimental model of small bowel transplantation. Our findings revealed a compelling association between reduced Tregs and the development of ACR in both patients and the animal model. Furthermore, experimental evidence suggested that Treg expansion within the small bowel mucosa might serve as a protective mechanism against histological damage. Consequently, therapeutic strategies aimed at maximizing regulatory circuits may improve ITx recipient outcomes by attenuating the allogenic response with fewer side effects than conventional immunosuppression.

## Materials and methods

### Clinical setting

#### Patients

This retrospective two-center study adhered to the principles outlined in the Declaration of Helsinki and received approval from the Ethics Committee (IRB: 1477–1119). Prior to the transplant, written consent was obtained for the utilization of patients’ blood samples and surgery specimens/biopsies for scientific proposes. Data were analyzed and summarized between January and August 2023 by the medical teams to safeguard patients’ anonymity.

Blood samples were obtained from 13 pediatric patients who underwent ITx at La Paz University Hospital (Madrid-Spain) between 2003 and 2018: 8 with normo-functional grafts (non-rejection group) and 5 displaying clinical, endoscopic, and histological features of ACR (rejection group). Small bowel biopsies and surgical specimens were collected from 10 pediatric patients who underwent ITx at Fundación Favaloro University Hospital (Buenos Aires-Argentina) between 2008 and 2019: 8 samples from individuals with normo-functional grafts and 3 from those confirmed with ACR. Importantly, both blood and grafts samples obtained from patients with active rejection were taken at the time of diagnosis, ruling out any potential confounding effects of drugs used to control the inflammatory process on the Treg subset. Detail patients’ information is depicted in Tables 1 and 2. The patients´ immunization status before the transplant is shown in S1 Table.

**Table 1:** Patients clinical features (n = 23).

Pt#	Recipient	Gender	Indication for the Tx	Graft Type	Induction	HLA-A/B/DR	Pre-Tx DSA	*De novo* DSA
	Age (years)*				Immunosuppression	Mismatches		
**1**	8,8	M	Retransplantation	MVTx	PP+IVIG+RTX	222	Class I and II	None
**2**	2,5	F	Dysmotility disorder	MVTx	CE+BAS	222	None	Class I
**3**	18,0	M	Retransplantation	MVTx	ALTZ	222	None	Class I and II
**4**	6,8	M	Retransplantation	MVTx	ALTZ	11	None	Class II
**5**	1,1	M	IFALD	SBTx-liver	CE+BAS+AZA	221	None	None
**6**	2,1	M	IFALD	SBTx-liver	TMG	N/A	None	None
**7**	3,2	F	IFALD	MVTx	CE+BAS	221	None	None
**8**	2,8	M	IFALD	SBTx-liver	TMG	210	None	None
**9**	16,2	F	IFALD	MVTx	ALTZ	122	None	Class II
**10**	6,4	M	Retransplantation	SBTx	CE+BAS	111	None	Class I and II
**11**	3,3	F	Tumor	MVTx	CE+BAS	222	None	None
**12**	9,7	M	IFALD	MVTx	CE+BAS	112	None	Class I and II
**13**	6,6	M	Retransplantation	MVTx	CE+BAS	222	None	None
**14**	15,7	M	Catheter related sepsis	SBTx	BAS	211	None	None
**15**	12,3	M	Catheter related sepsis	SBTx	TMG	222	None	None
**16**	16,3	M	Retransplantation	SBTx	TMG	222	N/A	N/A
**16**	16	M	Retransplantation	SBTx	TMG	222	N/A	N/A
**17**	3,1	F	Lack of vascular access	SBTx	BAS	N/A	None	Class I and II
**18**	2,8	F	Lack of vascular access	SBTx	BAS	220	None	Class I and II
**19**	2,8	F	Lack of vascular access	SBTx	BAS	211	None	Class I and II
**20**	17	M	Catheter related sepsis	SBTx-liver + kidney	TMG, PP, RTX, IVIG	111	None	None
**21**	0,8	F	IFALD	SBTx-liver	BAS	N/A	None	Class I and II
**22**	10,8	F	Lack of vascular access	SBTx	TMG, BAS	122	None	Class II
**23**	4,3	M	Lack of vascular access	SBTx	BAS	211	None	None

**Tx:** Transplant

**IFALD:** Intestinal failure-associated liver disease

**SBTx:** Isolated small bowel transplantation

**SBTx-Liver:** combined isolated small bowel transplantation + liver

**MVTx:** Multivisceral transplantation

**DSA:** Donor specific antibodies

**ALTZ:** Alemtuzumab

**PP:** Plasmapheresis

**IVIG:** intravenous immunoglobulin

**RTX:** Rituximab

**CE:** Corticosteroids

**BAS:** Basiliximab

**AZA:** Azathioprine

**TMG:** Thymoglobulin

**N/A:** No available data

* at the time of sample collection

**Table 2:** Incidence of rejection, laboratory analysis for Treg detection, immunosuppression, and DSA at the time of analysis.

Pt#	Confirmed	Laboratory	Time After Tx for Sample	Immunosuppression at the	DSA at time of study
	ACR	Analysis	Collection (years)	Time of Sample Collection	
**1**	No	FC	7,2	Tac + RAPA	Negative (clearance of preformed DSA)
**2**	No	FC	1,8	Tac + RAPA	Positive
**3**	No	FC	1,8	Tac + RAPA	Negative
**4**	No	FC	5,0	Tac + RAPA	Negative (clearance after antibody removal therapy)
**5**	No	FC	16,8	Tac	Negative
**6**	No	FC	11,9	Tac	Negative
**7**	No	FC	8,5	Tac	Negative
**8**	No	FC	15,3	Tac	Negative
**9**	Yes	FC	1,6	Tac + RAPA	Negative (dnDSA appeared lately)
**10**	Yes	FC	11,9	Tac	Negative (dnDSA appeared lately)
**11**	Yes	FC	0,3	Tac	Negative
**12**	Yes	FC	4,2	Tac	Negative (clearance after antibody removal therapy)
**13**	Yes	FC	8,0	Tac	Negative
**14**	Yes	IHC	3,7	Tac + RAPA	Negative
**15**	Yes	IHC	3,1	Tac + RAPA	N/A
**16**	Yes	IHC	1,2	Tac + RAPA	N/A
**16**	No	IHC	1,4	Tac + RAPA	N/A
**17**	No	IHC	0,9	Tac + RAPA	N/A
**18**	No	IHC	8,7	Tac + RAPA	N/A
**19**	No	IHC	8,8	Tac + RAPA	N/A
**20**	No	IHC	0,3	Tac + RAPA	Negative
**21**	No	IHC	5,4	Tac	N/A
**22**	No	IHC	0,1	Tac	N/A
**23**	No	IHC	3,1	Tac	Negative

**IHC:** Immunohistochemistry

**FC:** Flow cytometry

**Tac:** Tacrolimus

**RAPA:** Rapamycin

**DSA:** Donor specific antibodies

#### Flow cytometry analysis

Blood samples were gathered between 2018–2020 and analyzed within 24h after collection. Frequency and absolute number of lymphocytes, T-cells, CD4^+^ T-cells and Tregs were determined using the BD Multitest^TM^ CD3/CD16 + CD56/CD45/CD4/CD19/CD8 kit according to the manufacturer’s instructions. Additionally, anti-HLA-DR (Alexa Fluor 750), anti-CD45RA (PE-Cy7), anti-CD25 (PE, Becton Dickinson), and anti-CD127 (APC, Becton Dickinson) antibodies were added to the mix. Lymphocytes were identified based on their forward/side scatter characteristics, and doublets excluded following a forward height vs. forward area analysis. T-cells were identified as CD45^+^CD3^+^; and subsequently differentiated into CD4^+^ and CD8^+^ subsets. Regulatory T-cells were defined as CD45^+^CD3^+^CD4^+^CD25^high^CD127^-^ [18]. FoxP3 intracellular staining was performed to confirm the Treg phenotype. Additionally, CD19 was used to exclude B-cells from the analysis, and CD16/CD56 markers utilized to identify both natural killer (NK) and NKT cells. Data analysis was conducted using FlowJo v10 software (TreeStar, Ashland, OR, USA).

#### FoxP3 immunohistochemistry

Waxed graft samples were sectioned into five-μm slides and affixed onto positively charged glasses for deparaffinization and rehydration. Subsequently, antigen retrieval was performed using a 1mM ethylene-diamine-tetra-acetic buffer (pH = 9) and microwave treatment. Endogenous peroxidase activity was blocked using 3% H_2_O_2_/methanol solution.

For Treg detection, samples were incubated for 1 h at 37°C with a mouse anti-FoxP3 monoclonal antibody (clone 236A/E7; Abcam), followed by another 1 h incubation with a goat anti-mouse IgG antibody conjugated to horseradish peroxidase (DAKO, Carpinteria, CA, USA). Finally, FoxP3 positive cells were detected using a DAB kit (DAKO, Carpinteria, CA, USA). For each sample, FoxP3-positive cells were counted in five randomly selected fields at 40X magnification. Finally, results were averaged and presented as a single value per sample.

#### Human Leukocyte Antigen typing

Human Leukocyte Antigen (HLA)-A, -B, and DR were typed from all recipients and for 21 out of the 23 donors. DNA samples were isolated from peripheral blood mononuclear cells and the allelic variants analyzed by microlymphocytotoxicity (Lambda Monoclonal Typing Tray Set, One Lambda, USA) until 2015 conventional. Thereafter, sequences were typed with specific primers (Micro SSP^TM^ Generic HLA Class I DNA Typing Tray, One Lambda, USA). HLA-DR typing was always performed with sequence specific primers (Micro SSP^TM^ Generic HLA Class II DNA Typing Tray, One Lambda, USA).

#### Anti-HLA antibodies

Serum samples were collected from 22 out of the 23 enrolled patients. The presence of anti-HLA antibodies was assessed by Luminex (LABScreen mix kit, One Lambda, CA, USA) as previously reported by Talayero and collaborators [19]. A standard fluorescence intensity exceeding 15.000 or 20.000 was considered positive for anti-HLA class I and class II antibodies, respectively.

## Experimental model

### Animal use and care

Male adult Brown Norway (major histocompatibility complex I, MHC I: RT1^n^) and Lewis (MHC I: RT1^l^) rats weighing 180–250 g were purchased from Charles River (Saint Germain Nuelles-France) and housed individually at IdiPAZ animal facilities with a 12 h light-dark cycle. The room temperature was 21 ± 2°C and the relative humidity of 45 ± 15%. This study was performed in strict accordance with the European Union *Criteria for Animal Use in Scientific Experimentation* (63/2010) and the Spanish legislation (RD 53/2013). The protocol was approved by the local animal Welfare Ethics Committee (PROEX 59.8/2020).

### Heterotopic intestinal transplantation procedures

Heterotopic allogenic ITx from Brown Norway (donor) to Lewis (recipient) was performed as previously described [20, 21]. Briefly, the allogenic small bowel (jejunum and ileum) was engrafted using two end-to-side vascular anastomoses: donor portal vein to recipient´s cava vein, and donor superior mesenteric artery was left with an aortic cuff, which was anastomosed to the recipient´s abdominal aorta. The proximal lumen end was closed, and an ileostomy was performed with the distal end. The graft was positioned in the right side of the abdominal cavity, while the native bowel remained in place. To prevent hypothermia, animals were placed on a thermal blanket throughout the surgical procedure, with oxygen administrated at a rate of 1 L/h. Inhalation anesthesia consisted of 5% isoflurane for induction and 2–3% for maintenance. Subcutaneous buprenorphine (0.04 mg/kg) was administered as analgesic before starting the procedure and every 12 h for 3 days after transplantation. Additionally, local anesthesia (lidocaine 2%) was administered subcutaneously in the surgical incision and stoma.

### Recipient and graft clinical monitoring

Recipient clinical monitoring was conducted daily to evaluate body wight, graft tightening, ocular secretions, hair appearance, posture, attitude, and fecal production. These signs were quantified and treated based on preestablished criteria [20], detailed information is provided in Table 3. Each recipient received a final clinical score derived from the sum of the individual parameter. The humanitarian endpoint was applied under the following circumstances: total clinical score equaled or exceeded 4, weight loss exceeding 20% of the recorded value after transplantation, or detection of graft hardening.

**Table 3:** Clinical status score.

Clinical parameter/severity	Graduation
Ocular/nose secretion	
Absence	0
Mild	1
Moderate	2
Severe	3
Piloerection	
Absence	0
Mild	1
Moderate	2
Severe	3
Antalgic posture	
Absence	0
Mild	1
Moderate	2
Severe	3
Lethargic attitude	
Absence	0
Mild	1
Moderate	2
Severe	3
Feces production	
Yes	0
No	1
Feces consistency	
Solid	0
Pasty	1
Liquid	2

### Experimental groups

After transplantation, animals were randomly assigned into one of the following groups (n = 5 rats per group): **1)** without immunosuppressant treatment (w/o IS): no pharmacological intervention was administered; **2)** rapamycin treatment: daily oral 2 mg/kg doses until the endpoint; **3)** tacrolimus treatment: daily subcutaneous 0.6 mg/kg doses until the endpoint; and **4)** tacrolimus treatment for 7 days: subcutaneous 0.6 mg/kg doses of tacrolimus for 7 days, followed by withdrawal of immunosuppressant treatment until the endpoint. Endpoints were scheduled for day 14 after surgery but advanced in case of significant clinical deterioration. Animals were euthanized by exsanguination under general anesthesia. Finally, immunosuppressant blood levels were assessed as previously described by our group [22, 23].

### Sample collection

Samples were collected at days 3, 7, and 14 after ITx (or at the humanitarian endpoint). Blood and graft samples were obtained through the tail vein and by peristomal dissection and segmental resection of the stoma, respectively. Control blood samples were obtained from Brown Norway non-transplanted animals and control small bowel tissue from donor rats during graft procurement.

### Isolation of lamina propria cells

Small bowel lamina propria cells were isolated as previously described [24], washed, and suspended in phosphate-buffered saline (PBS). T-cell subsets were analyzed by flow cytometry. Further details on this procedure are available in Supplementary Materials.

### Peripheral blood mononuclear cell isolation

Peripheral blood mononuclear cells were isolated by Ficoll–Hypaque gradient centrifugation (GE Healthcare Life Sciences, Little Chalfont, UK). After two washes with PBS, cells were re-suspended in sterile PBS and analyzed by flow cytometry.

### Flow cytometry analysis

Peripheral blood mononuclear cells and small bowel lamina propria cells were incubated for 30 min at 4°C with the following fluorochrome-conjugated antibodies: anti-CD3 (PerCP-Vio 700; Miltenyi Biotec), anti-CD4 (APC-Cy7; Biolegend), anti-CD8 (PE-Cy7; Biolegend), anti-CD25 (APC; Biolegend), anti-CD45 (Alexa 700; Biolegend), and anti-FoxP3 (Brilliant Violet 421; Biolegend). Blood and graft chimerism were assessed using FITC-conjugated antibodies targeting the Lewis and Brown Norway MHC class I molecules: anti-RT1^l^ (BD Biosciences, Franklin Lakes, NJ, USA) and anti-RT1^n^ (Bio-Rad, Hercules, CA, USA), respectively. For FoxP3 intracellular staining, cells were fixed and permeabilized using Fix/Perm buffer (Biolegend) according to the manufacturer’s instructions. Flow cytometry was performed on a Navios cytometer (Beckman Coulter, Brea, CA, USA), cells were gated according to their forward and side scatter characteristics. Data were analyzed using FlowJo v10 software (TreeStar, Ashland, OR, USA).

### Histopathological analysis

Histological examination was performed on 5-μm tissue sections stained with hematoxylin–eosin by an experienced pathologist, who was blinded to the sample identities. The histological alterations related to graft rejection were assessed using a semi-quantitatively score [25]. Detailed information is presented in Table 4.

**Table 4:** Histological criteria for grading small bowel allograft acute rejection.

Grade	Major histologic findings
	Minimal localized inflammatory infiltrate, minimal crypt epithelial injury,
Indeterminate ACR	< 6 apoptotic bodies/crypts, absence -minimal architecture distortion,
	no mucosal ulceration
	Mild localized inflammatory infiltrate with activated lymphocytes,
Mild ACR	mild crypt epithelial injury, > 6 apoptotic bodies/10 crypts,
	mild architecture distortion, no mucosal ulceration.
	Widely dispersed inflammatory infiltrate in lamina propria,
Moderate ACR	diffuse crypt epithelial injury, increased crypt apoptosis with focal
	confluent apoptosis, prominent architecture distortion, no mucosal ulceration.
Severe ACR	Features of moderate ACR plus mucosal ulceration, possible severe intimal
	arteritis or transmural arteritis may be seen.

**ACR:** acute cellular rejection

### Real-time RT-qPCR

The mRNA levels of several pro- and anti- inflammatory mediators, including monocyte chemoattractant protein one (MCP-1), tumor necrosis factor (TNF), interferon gamma (IFNγ), eosinophil chemotactic protein 11 (CCL-11), interferon gamma-induce protein 10 (CXCL-10), interleukin (IL)-6, IL-10, IL-13, IL-17, IL-22, indoleamine 2,3-dioxygenase (IDO), and transforming growth factor beta (TGFβ) were analyzed in small bowel samples using the comparative threshold (CT) method [26], with beta actin (β-actin) as the normalization control. Primers sequences are provided in Table 5. Data were expressed as relative gene expression (2^−ΔCt^). Detailed information on RNA extraction and real-time quantitative PCR is provided in the Supplementary Materials and Methods.

**Table 5:** Primers sequences.

Gene	Forward primer	Reverse primer
**MCP-1**	TCC ACA TTC GGA GGC TAA AG	ACG TGA AGG TTC AAG GAT GC
**IL-6**	CTG ATT GTA TGA ACA GCG ATG	GAA CTC CAG AAG ACC AGA GC
**TNFα**	CCACCAAGCGGAGGAGCAGC	TCGGCTGACGGTGTGGGTGA
**IFNγ**	TAC ACG CCG CGT CTT GGT	GAG TGT GCC TTG GCA GTA ACA G
**CCL-11**	CACCCAGGTTCCATCCCAAC	GCTTTCAGCGTGCATCTGTT
**CXCL-10**	CTGCACCTGCATCGACTTCC	TTCTTTGGCTCACCGCTTTC
**IL-13**	GCA ACA TCA CAC AAG ACC AGA AG	TGT CAG GTC CAC GCT CCA T
**IL17**	ACAGTGAAGGCAGCGGTACT	GCTCAGAGTCCAGGGTGAAG
**IL-22**	TGGTGCCTTTCCTGACCAA	GTTCTGGTCATCACCGCTGAT
**IL-10**	CGA CGC TGT CAT CGA TTT CTC	TCT TGG AGC TTA TTA AAA TCA TTC TTC
**IDO**	CAG ACA CCT TTT TCC ACG	CAG CAG ATC CTT CAC CAA CG
**TGFβ**	CTC AAC ACC TGC ACA GCT CC	ACG ATC ATG TTG GAC AAC TGC T
**β-actin**	ACAACCTTCTTGCAGCTCCTC	CTGACCCATACCCACCATCAC

**MCP-1**: monocyte chemoattractant protein one

**IL-6:** interleukin 6

**TNFα:** tumor necrosis factor alpha

**IFNγ:** interferon gamma

**CCL-11:** eosinophil chemotactic protein

**CXCL-10:** interferon gamma-induced protein

**IL-13**: interleukin 13

**IL-17:** interleukin 17

**IL-22:** interleukin 22

**IL-10:** interleukin 10

**IDO:** indoleamine 2,3-dioxygenase

**TGFβ:** transforming growth factor beta

**β-actin:** beta actin

### Serum levels of IL-6 and IL10

Plasma IL-10 and IL-6 levels were analyzed by Luminex (FLEXMAP 3D, Labclinics). Each sample was measured by duplicate, and then analyzed against standard curves following manufacturer’s instructions. Results are presented as ƞg/mL.

### Statistical analysis

Continuous variables are presented as mean ± standard error of the mean and were analyzed using Student’s *t*-test (paired or unpaired comparisons of two groups) or analysis of variance followed by Tukey’s test (multiple comparisons). Non-continuous variables were analyzed using Kruskal–Wallis or Mann–Whitney *U* test, as appropriate. Animal survival was compared using Kaplan–Meier curves with log-rank Mantel-Cox test. Relative gene expression was normalized and plotted on a Heat map, while principal component analysis was employed to detect similarities between groups [20, 27]. Statistical significance was defined as p < .05. Analyses were performed using GraphPad Prism 9.4.

## Results

### Clinical setting

#### Acute cellular rejection is associated with reduced number of regulatory T-cells

**Fig 1:**
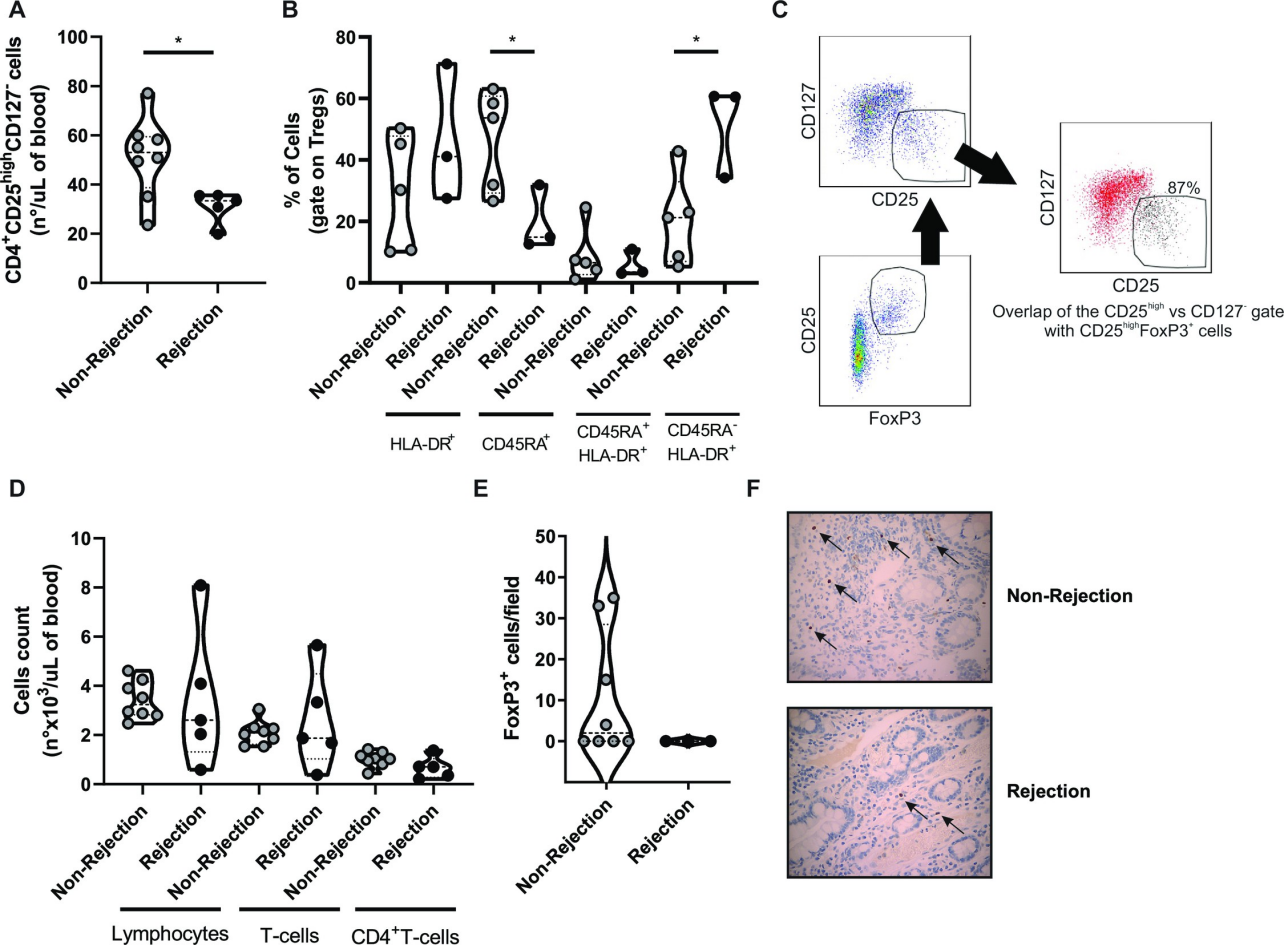
Regulatory T cell frequency is reduced in pediatric patients during intestinal cellular rejection. **(A)** CD4^+^CD25^high^CD127^-^ Treg frequency in blood of patients with and without graft rejection (n°/μL of blood; non-rejection n = 8, rejection n = 5). **(B)** Percentage of blood Tregs HLA-DR^+^, CD45RA^+^, CD45RA^+^HLA-DR^+^, and CD45RA^-^HLA-DR^+^ (non-rejection n = 5, rejection n = 3). **(C)** Representative dot plots showing that CD4^+^CD25^high^CD127^-^ blood lymphocytes express the FoxP3 intracellular marker (n = 3); CD25^high^FoxP3^+^ T-cells are depicted with black dots (right plot). **(D)** Frequency of lymphocytes, T-cells, and CD4^+^T-cells in blood samples of patients with and without graft rejection (n°x10^3^/μL of blood). **(E)** Number of FoxP3 cells per field in the small bowel lamina propria (non-rejection n = 8, rejection n = 3). **(F)** Representative graft immunohistochemistry’s of patients without rejection (upper panel) and with active rejection (lower panel), black arrows indicate the FoxP3 positive cells. Results are presented as violin plots with mean and SEM (dotted lines). *p < .05.

Regulatory T-cells have been widely described as key players of gut homeostasis [8, 9]. However, their involvement in the allogenic response to intestinal grafts remains uncertain. Therefore, we initially assessed the Treg frequency in blood and graft samples from pediatric patients by flow cytometry (CD4^+^CD25^high^CD127^-^ cells) and immunohistochemistry (FoxP3 staining), respectively. As depicted in Fig 1A, the absolute number of blood Tregs was significantly lower in patients undergoing rejection compared to those with normo-functional grafts (p < .05), and displayed a less naïve (lower percentage of CD45RA^+^ cells, p < .05) and more activated/memory (higher percentage of CD45RA^-^HLA-DR^+^ cells, p < .05) phenotype (Fig 1B). Of note, CD4^+^CD25^high^CD127^-^ cells co-express the intracellular marker FoxP3 (Fig 1C), and no differences in frequency of total lymphocytes, T-cells or CD4^+^T-cells was observed between patients with or without graft rejection (Fig 1D). In the small bowel mucosa, we also observed a tendency toward a reduced FoxP3 cells count per field in tissue samples from inflamed areas compared to those obtained from patients without graft rejection; however, due to data dispersion, the differences were not statistically significant (Fig 1E and 1F). No correlation between Treg count and conditioning immunosuppression, number of HLA mismatches or DSA was observed (S1 Fig).

Considering the limited number of patients enrolled in our study, the cohort´s diversity, and the complexity of the immunosuppressive protocols in the clinical setting, we decided to use a rat model of small bowel transplantation to further study the association between Tregs and the rejection phenomena.

## Experimental model

### First-line treatment with tacrolimus is more efficient than rapamycin in protecting against acute cellular rejection

**Fig 2:**
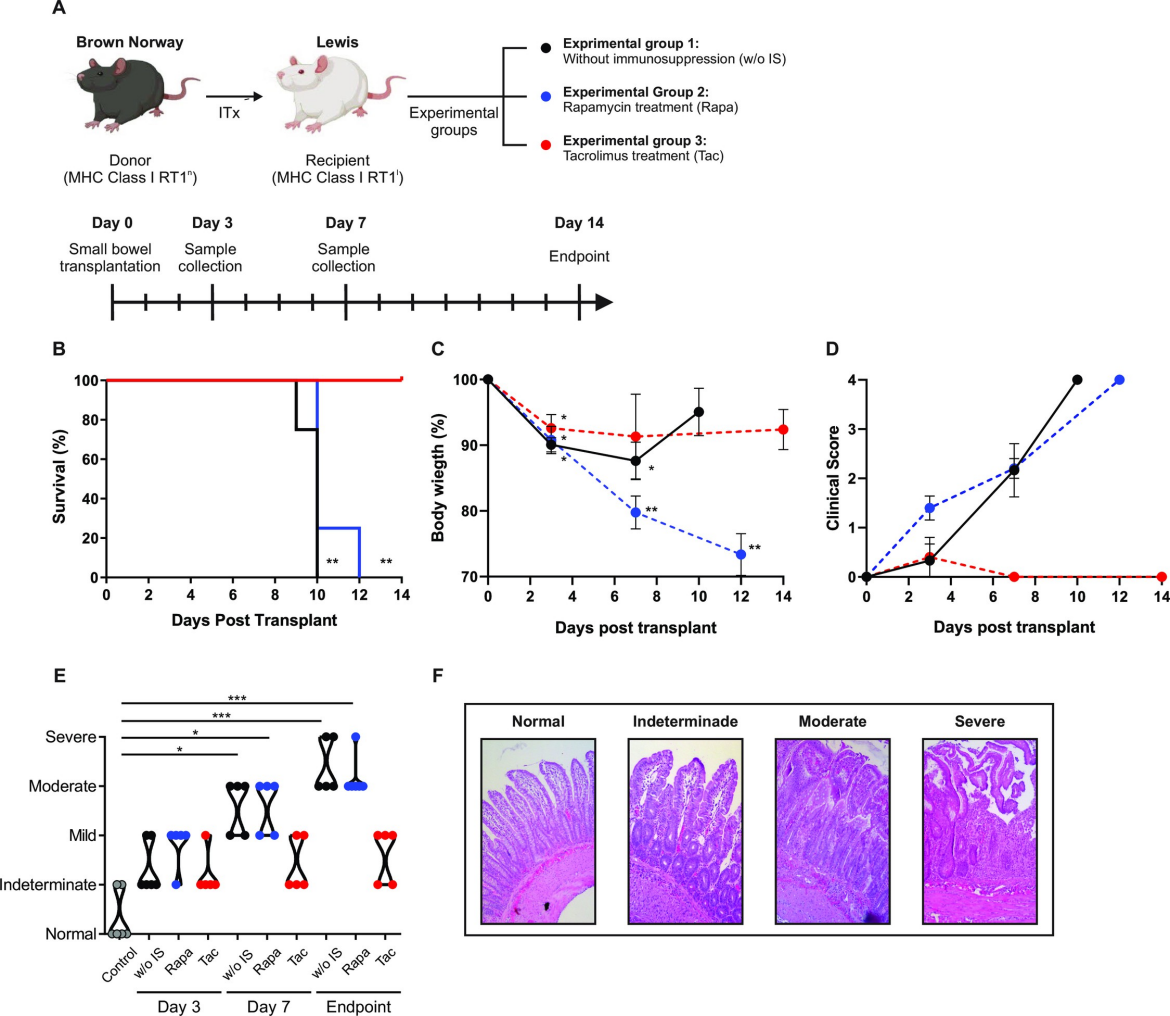
Survival, clinical and histological signs of acute cellular rejection in intestinal graft recipients. **(A)** Flow diagram of the experimental model. **(B)** Survival curves of intestinal graft recipients. **(C)** Percentage of body weight loss compared with day 0 after transplant. **(D)** Recipients clinical score from day 0 after transplant until de endpoint. **(E)** Histological score of ACR in control samples (before implanting the graft) and at day 3, 7, 14 or endpoint. **(F)** Microscopic graft appearance in representative cases of normal histology or indeterminate, mild moderate and severe graft ACR. Results are presented as mean ± SEM or as violin plots with mean and ESM (dotted lines). Lines/dots: Black, animals without immunosuppressive treatment (w/o IS); Blue, animals treated with 2mg/kg/day of rapamycin (Rapa); Red, animals treated with 0,6mg/kg/day of tacrolimus (Tac); Grey, graft samples taken before implantation (control). In all cases n = 5/group. *p < .05; ** p < .01; *** p < .001.

To simplify the pharmacological protocols used with the patients we evaluated the clinical status and histopathological signs of ACR in a ITx model where recipients were treated only with rapamycin or tacrolimus, two of the most relevant immunosuppressive drugs in the clinical settings [28, 29]. A flow diagram illustrating the rat strains used for the transplant, the experimental groups, and the days of sampling is depicted in Fig 2A. Consistent with our previous findings [20], animals without immunosuppressant treatment were sacrificed between days 9 and 10 post-transplantation (Fig 2B). Although recipients did not exhibit significant weight loss at the endpoint (Fig 2C), they display marked clinical deterioration (Fig 2D) and moderate to severe ACR histological scores (Fig 2E). Importantly, histopathological findings showed significant graft rejection by day 7 post-transplant. In contrast, animals treated with tacrolimus reached day 14 without clinical deterioration (Fig 2B–2D) or significant histological changes in the graft (Fig 2E). As previously demonstrated in an orthotropic model [28], animals under rapamycin treatment experienced significant weight loss and clinical deterioration between days 10–12 (Fig 2B–2D). Regardless the use of this drug, animals exhibited histopathological findings of mild to moderate ACR by day 7 after transplantation, and moderate to severe changes at the time of sacrifice (Fig 2E). Notably, rapamycin blood levels at the endpoint ranged from 4.7 to 7.9 ng/mL (S2A Fig), indicating therapeutic levels of immunosuppression. Representative images of the normal small bowel histology and indeterminate, mild, moderate, and severe ACR are provided in Fig 2F.

### Graft rejection is associated with increased CD8 T-cell frequency in blood and loss of chimerism

**Fig 3:**
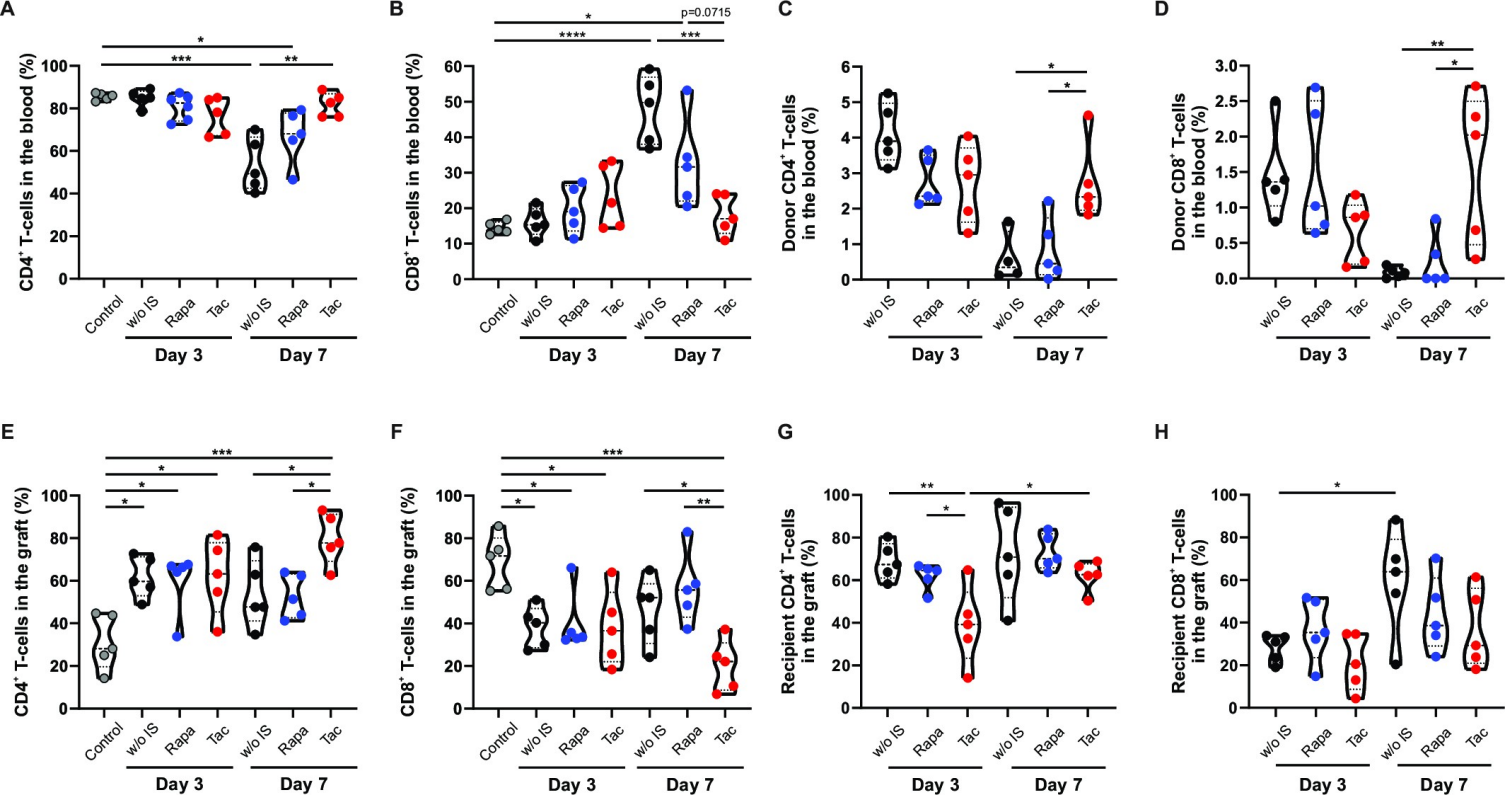
Frequency of CD4 and CD8 T-cell in blood and graft after transplant. **(A)** CD4 T-cell frequency in blood samples of control animals and animals without immunosuppression treatment, rapamycin treatment, and tacrolimus treatment at days 3 and 7 after transplant. **(B)** CD8 T-cell frequency in blood samples of control animals and animals without immunosuppression treatment, rapamycin treatment, and tacrolimus treatment at days 3 and 7 after transplant. **(C)** Frequency of donor CD4 T-cells (BN MHC I^+^ CD4^+^) in the blood at days 3 and 7 after transplant. **(D)** Frequency of donor CD8 T-cells (BN MHC I^+^ CD8^+^) in the blood at days 3 and 7 after transplant. **(E)** CD4 T-cell frequency in graft samples of control animals and animals without immunosuppression treatment, rapamycin treatment, and tacrolimus treatment at days 3 and 7 after transplant. **(F)** CD8 T-cell frequency in graft samples of control animals and animals without immunosuppression treatment, rapamycin treatment, and tacrolimus treatment at days 3 and 7 after transplant. **(G)** Frequency of recipient CD4 T-cells (LEW MHC I^+^ CD4^+^) in the graft at days 3 and 7 after transplant. **(H)** Frequency of recipient CD8 T-cells (LEW MHC I^+^ CD4^+^) in the graft at days 3 and 7 after transplant. LEW: Lewis; BN: Brown Norway; w/o IS: Animals without immunosuppressant treatment (black dots); Rapa: Rapamycin treatment (blue dots); Tac: Tacrolimus treatment (red dots); grey dots: control samples. Results are presented as violin plots with mean and SEM (dotted lines). *p < .05; **p < .01; *** p < .001; **** p < .0001.

Considering that changes in the frequency of CD4 and CD8 T-cells have been associated with the rejection phenomena, we aim to determine whether our experimental model could also recapitulate these findings. Gating strategy is shown in S2B Fig. As depicted in Fig 3A and 3B, the initial stage of ACR was accompanied by a significant decrease in the blood frequency of CD4 T-cell (p < .001 and .05, respectively) and concomitant increase in the percentage of CD8 lymphocyte, compared to that in the control animals (p < .0001 and .05, respectively). In contrast, neither the CD4 nor CD8 subsets showed changes in tacrolimus-treated animals (Fig 3A and 3B). On the other hand, persistent blood chimerism has been highlighted as indicative of a “harmonic” interaction between donor and recipient immune cells [30–32]. Consistent with these findings, the percentage of donor CD4 and CD8 T-cells in blood exhibited a significant decreased only in groups diagnosed with ACR (Fig 3C and 3D; p < .01 and .05, respectively). These data indicate that, in our animal model, histological evidence of ACR was associated with an increased frequency of circulating CD8 T-cells and loss of blood lymphocyte chimerism.

### Small bowel lamina propria repopulation by recipient T-cells is faster in intestinal allografts that end in acute cellular rejection

Next, we focused on the graft´s immunological responses following transplantation. As described by Talayero et al. [33], the frequency of CD4 T-cell was rapidly increased in the small bowel mucosa, irrespective of the immunosuppressant treatment (Fig 3E, p < .05). These findings were also accompanied with a diminished CD8 percentage within the graft (Fig 3F, p < .05). Interestingly, as observed in the blood samples, animals with ACR showed a heightened CD8 frequency in the graft at day 7 post-transplantation, compared to that in the tacrolimus group (animals w/o IS treatment: p < .05; animals with rapamycin treatment: p < .01; Fig 3F).

Analysis of the graft cell repopulation revealed a rapid influx of recipient T-cells into the lamina propria (Fig 3G and 3H). By day 3 after transplantation, 68.7 ± 3.9% of the CD4 subset consisted of recipient cells in animals without immunosuppressant treatment, with no significant differences compared to rapamycin-treated group (61.7 ± 2.7% of total CD4 lymphocytes were recipient cells). In contrast, the tacrolimus-treated group exhibited a significantly lower percentage of recipient CD4 T-cells at day 3 (38.9 ± 8.2%) compared to animals without immunosuppressant treatment (p < .01) and those receiving rapamycin (p < .05). By day 7 after transplantation, regardless of the presence/absence of graft ACR, the recipient CD4 T-cell frequency did not differ significantly between the three experimental groups (animals without immunosuppressant treatment: 72.6 ± 10.3%; rapamycin-treated animals: 73.1 ± 3.8%; tacrolimus-treated animals: 62.1 ± 3.2%). Interestingly, as shown in Fig 3G and 3H, the recipient´s CD8 frequency in the graft was notably lower than that observed for CD4 lymphocytes (day 3: 33.8 ± 3.8%, 36.8 ± 6.7%, and 21.4 ± 6%, in the untreated, rapamycin, and tacrolimus groups, respectively; day 7: 59.3 ± 11.2%, 43.7 ± 8%, and 36.7 ± 8.3 in the untreated, rapamycin, and tacrolimus groups, respectively). These findings highlight the greater ability of CD4 T-cells to infiltrate the graft compared to CD8 lymphocytes, potentially explaining the increased CD4 percentage after transplantation compared to that in the control samples. Furthermore, first-line treatment with tacrolimus may facilitate a more gradual replacement of the T-cell compartment in the early post-transplant stages, enabling regulatory mechanisms to adapt and control the allogenic response.

### Acute cellular rejection is associated with decreased Treg frequency

**Fig 4:**
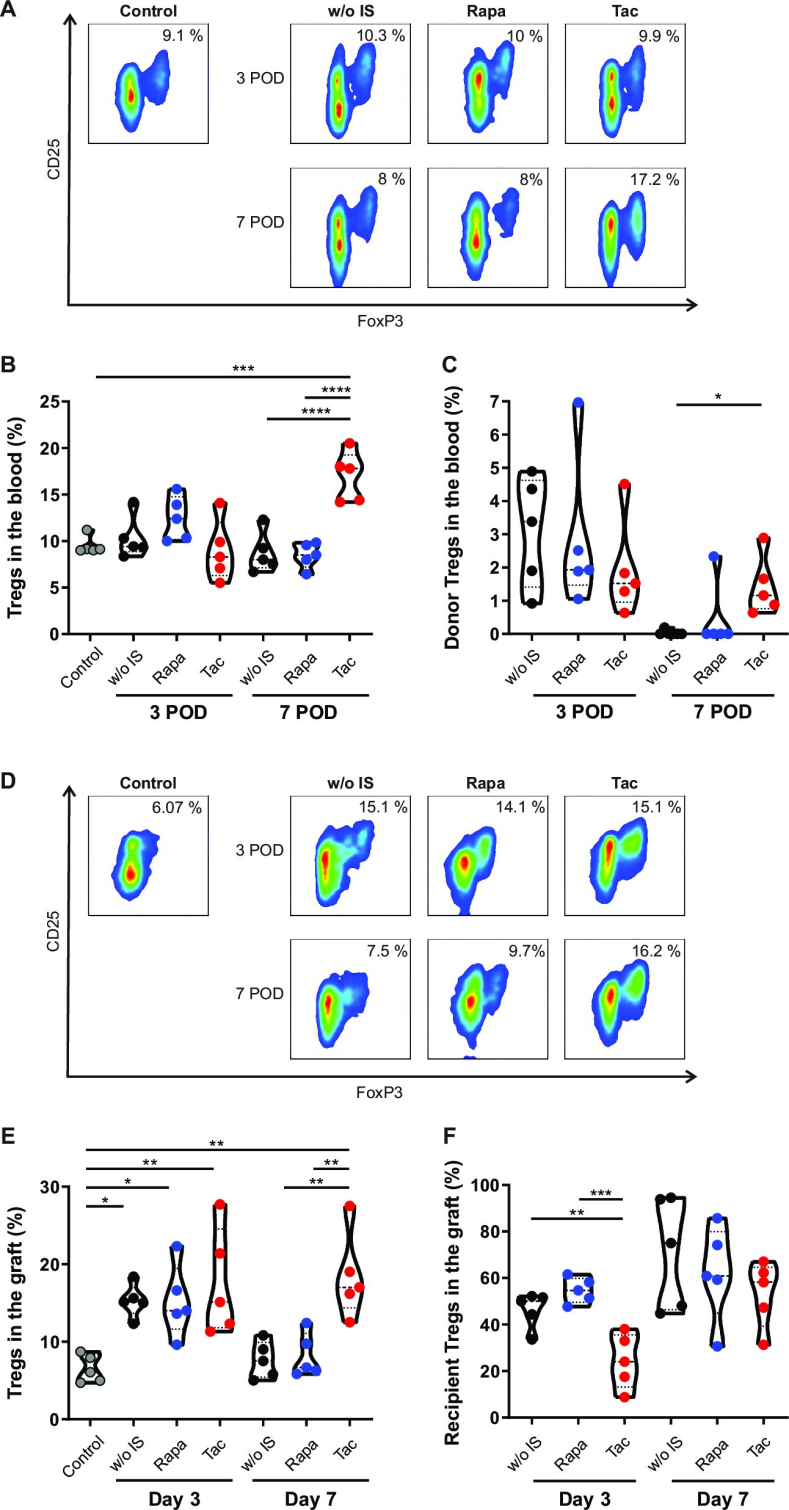
Frequency of regulatory T-cells in blood and graft. **(A)** Representative dot plots depicting Treg frequency in blood samples from control animals and those without immunosuppressant treatment, rapamycin treatment and tacrolimus treatment at days 3 and 7 after transplant. **(B)** Treg frequency in blood samples from control animals and animals without immunosuppressant treatment, rapamycin treatment and tacrolimus treatment at days 3 and 7 after transplant. **(C)** Donor Treg frequency (BN MHC I^+^) in blood at days 3 and 7 after transplant. **(D)** Representative dot plots depicting Treg frequency in graft samples from control animals and samples from animals without immunosuppressant treatment, rapamycin treatment and tacrolimus treatment at days 3 and 7 after transplant. **(E)** Treg frequency in graft samples from control animals and animals without immunosuppressant treatment, rapamycin treatment and tacrolimus treatment at days 3 and 7 after transplant. **(F)** Recipient Treg frequency (LEW MHC I^+^) in graft samples at days 3 and 7 after transplant. LEW: Lewis; BN: Brown Norway; w/o IS: Animals without immunosuppressant treatment (black dots); Rapa: Rapamycin treatment (blue dots); Tac: Tacrolimus treatment (red dots). Grey dots: control samples. Results are presented as violin plots with mean and SEM (dotted lines). *p < .05; **p < .01; *** p < .001, ****p <, 0001.

As shown in Fig 4A and 4B, the frequency of blood Tregs remained stable despite graft ACR (animals without immunosuppression and those treated with rapamycin). However, recipients treated with tacrolimus exhibited a significant increase in the blood CD4^+^CD25^high^FoxP3^+^ percentage by day 7 compared to control animals (p < .001) and those with active ACR (p < .0001). Notably, only tacrolimus-treated animals conserved donors´ Tregs in the blood 7 days post-transplantation (Fig 4C).

In the graft, the frequency of CD4^+^CD25^high^FoxP3^+^ cells was heightened in all groups at day 3 compared to that in control samples (Fig 4D and 4E, p < .05), with this cell subset comprising both donor and recipient cells (Fig 4F), suggesting that regulatory circuits are also activated in the early stages of the alloreactive response. Interestingly, by day 7 post-transplantation, the CD4^+^CD25^high^FoxP3^+^ frequency in both the blood and mucosa only remained above normal levels when ACR was effectively inhibited by tacrolimus. Based on these findings, we investigate whether a high Treg frequency might correlate with beneficial effects on graft homeostasis.

### Increased Treg frequency following tacrolimus treatment was associated with graft protection after withdrawing the immunosuppression

**Fig 5:**
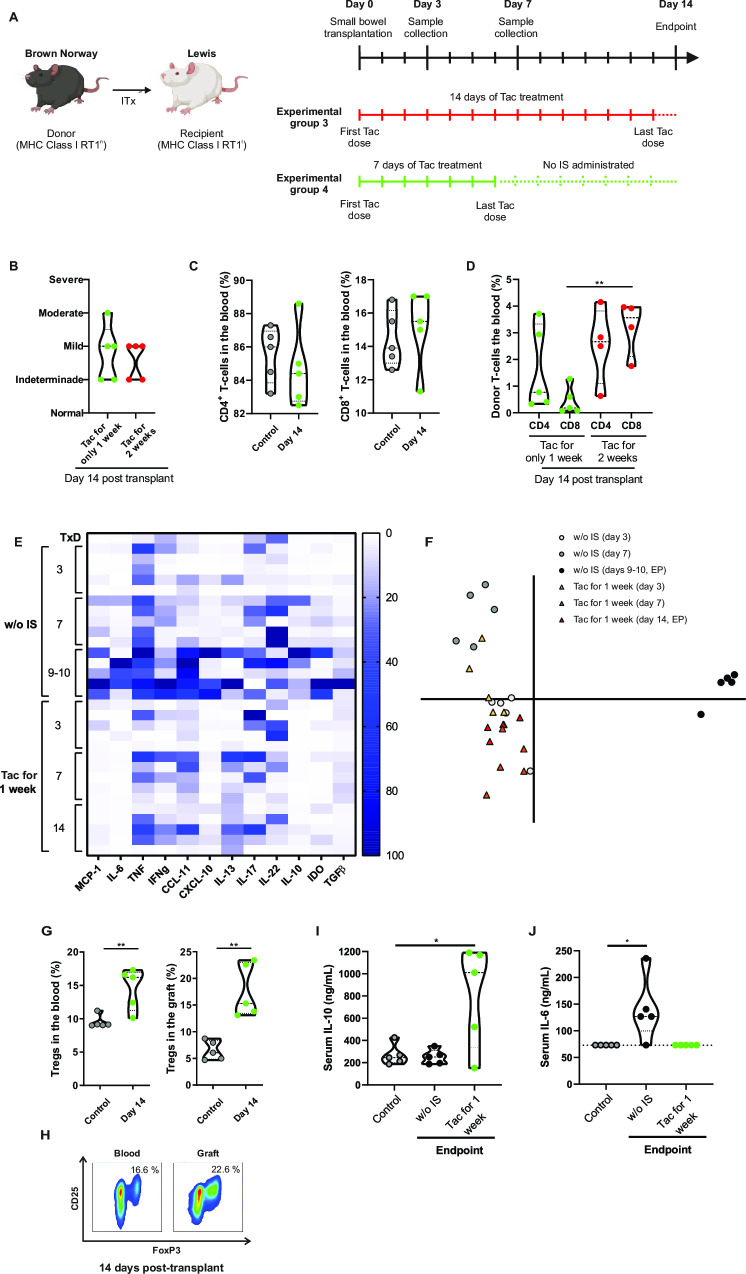
Increase frequency of Treg is associated with graft protection against acute cellular rejection. **(A)** Flow diagram of the experimental group. **(B)** Histological score of ACR at the endpoint in animals treated with tacrolimus during 14 consecutive days and those treated with tacrolimus for only 7 of the 14 days. **(C)** Frequency of CD4 and CD8 cells in blood of control animals and at day 14 after transplant in animals treated with tacrolimus for only 7 days. **(D)** Donor CD4 and CD8 T-cell frequency at day 14 post-transplant. **(E)**. Heat map showing mRNA relative expression of MCP-1, IL-6, TNF, IFNγ, CCL-11, CXCL-10, IL-13, IL-17, IL-22, IL-10, IDO and TGFβ in small bowel control samples (before engraftment), samples from animals without immunosuppression treatment, and graft samples of animals receiving Tac for only 7 days. **(F)** Principal component analysis of mRNA gene expression. **(G)** Percentage of Tregs in blood and graft in control samples and at day 14 after transplant in animals that receive tacrolimus for only 7 days. **(H)** Representative dot plot of the Treg frequency in blood and graft at day 14 in animals that receive tacrolimus for only 7 days. **(I)** and **(J)** Serum IL-10 and IL-6 levels in control samples, and samples taken at the endpoint in animals without immunosuppression treatment and tacrolimus treatment for only 1 week. Red dots: Tacrolimus treated animals; grey dots: control samples; green dots: Tacrolimus for 7 days followed by 7 days without immunosuppressant treatment. Tac: Tacrolimus. w/o IS: without immunosuppression. EP: endpoint. Dotted line: IL-6 detection limit. Results are presented as violin plots with mean and SEM (dotted lines). *p < .05, **p < .01.

Based on the observed increase in Treg frequency after 7 days of tacrolimus treatment, we investigated whether these cells could exert a protective effect against ACR. Therefore, we introduced a new experimental group where intestinal graft recipients received tacrolimus for 7 days to allow the expansion of CD4^+^CD25^high^FoxP3^+^ cells, followed by withdrawal of immunosuppression. Flow diagram of this group is shown in Fig 5A. Since animals receiving no immunosuppressant treatment developed histological findings indicative of intestinal ACR by day 7 after transplant (Fig 2E), recipients were sacrificed 7 days after suspending tacrolimus administration (day 14 post-transplantation). Furthermore, as depicted in Fig 3G and 3H, grafts are highly infiltrated with recipient T-cells by day 7 post-transplant, suggesting that the allogenic response may occur even more rapidly than that in animals not treated with immunosuppressant. Finally, to rule out the possibility of residual immunosuppression inhibiting the rejection process, we assessed tacrolimus blood levels at days 3, 7, and 9 after transplantation (S2C Fig). As expected, the tacrolimus levels decreased below the detection limit by day 9, indicating complete metabolism of the immunosuppressant. Interestingly, no significant histological changes were observed at day 14 between animals treated with daily doses of tacrolimus until the endpoint and animals treated with tacrolimus for only 7 days (Fig 5B). Moreover, at day 14 after transplantation, this new experimental group showed no changes in blood CD4 or CD8 frequency compared to the control values (Fig 5C), and still preserved blood chimerism (Fig 5D).

To reinforce the histological findings, we analyzed the mRNA expression levels of several pro-inflammatory mediators in the graft, comparing them with control samples and samples from animals with ACR (no immunosuppression group). As shown in the Heat map (Fig 5E), graft rejection was associated with up-regulation of MCP-1, IL-6, TNF, CCL-11, and CXCL-10 mRNA levels. In line with the histopathological findings, we observed no significant increase in the expression of any of these genes after withdrawing the pharmacological treatment (Fig 5E). In addition, principal component analysis showed that samples obtained at days 3, 7 and 14 post-transplant in animals treated with tacrolimus for only 1 week clustered together and separated from those obtained from animals with ACR (Fig 5F). Additionally, mRNA expression correlates with disease activity, with samples exhibiting mild/moderate rejection (day 7 post-transplant in animals with no immunosuppressant treatment) and moderate/severe rejection (days 9–10 post-transplant in animals with no immunosuppressant treatment) forming distinct clusters.

Finally, in this final experimental group, the Treg frequency at day 14 remained significantly up-regulated in both blood and graft samples compared to controls (Fig 5G and 5H). To elucidate potential regulatory mechanisms involved in graft protection, we analyzed the mRNA expression levels of several anti-inflammatory mediators in the mucosa but found no significant up regulation in any of them (Fig 5E). However, serum IL-10 levels were found to be significantly increased by day 14 post-transplantation in animals treated with tacrolimus for only one week, but similar to controls in animals not treated with immunosuppressants (Fig 5I). As expected, at the endpoint IL-6 serum levels were significantly increased in animals that did not receive immunosuppressive treatment, but similar to controls in animals treated with tacrolimus for 1 week (Fig 5J). These findings suggest that Tregs could be exerting their protective role against ACR through IL-10 secretion.

## Discussion

Tregs have emerged as key players in achieving a harmonious host/graft coexistence following transplantation [13, 15, 34, 35], with several studies linking their frequency to graft “operational tolerance” or rejection [10, 11, 15, 16]. However, in the field of ITx, where the incidence of rejection is significantly higher compared to other solid organ transplants [1–4], and the number of cases much more limited, this particular cell subset has been poorly evaluated. Therefore, we initially studied the Treg frequency in both blood and graft samples of pediatric patients and found that the ACR phenomena is associated with a reduction of this cell subset in liver containing grafts as well as in isolated intestinal transplant patients. To confirm these findings and gain further insight into the Treg dynamic upon rejection, we used a rat heterotopic small bowel transplantation model. This experimental approach enabled us to examine donor and recipient Treg migration, kinetics of expansion before and after ACR appearance, and its correlation with the allograft outcome at both molecular and histological levels.

Following the transplant animals were untreated or treated with either rapamycin or tacrolimus, two of the most frequently employed immunosuppressants in the clinical practice. Clinical deterioration and histological findings consistent with ACR were detected by day 7 after transplantation in both untreated and rapamycin-treated recipients, as previously reported in an orthotopic model of ITx [28]. Notably, rapamycin treated animals exhibited a more pronounced weight loss and clinical deterioration, likely associated with the reported wound healing interference of this immunosuppressant [36] and subsequent exacerbation of the immunological response triggered by ischemia-reperfusion injury [37]. Therefore, a preliminary phase of tacrolimus seems to be necessary in order to achieve the long-term benefits of mTOR inhibitors [38].

In our experimental model, the rejection phenomenon was accompanied by increased blood CD8/CD4 ratio, loss of T-cell chimerism, and upregulation of several pro-inflammatory genes in the graft mucosa. Despite their low sensitivity and specificity, these cellular events have been correlated with ACR [30–32, 39, 40] and poor survival rate in the clinical setting [41]. Furthermore, as reported for ITx patients [42], immunological monitoring of the graft showed a correlation between increased frequency of CD8 T cells and ACR, however we cannot assure their cytotoxic nature due to limitations of the model. Al these data together shows that our experimental model mirrors several cellular events already described in clinical practice, validating their combined use as markers of ACR.

Regarding Treg, we observed a physiological expansion of mucosal CD4^+^CD25^high^FoxP3^+^ lymphocytes by day 3 post-transplantation in all experimental groups, probably associated with the high influx of CD4 T-cells to the lamina propria. However, these heightened levels declined by day 7 in recipients diagnosed with ACR, likely as part of the allogenic response. Notably, tacrolimus-treated animals maintained their blood and tissue Treg frequencies above normal levels. These finding may seem contradictory, considering previous descriptions of tacrolimus as more detrimental to Tregs than rapamycin [43]. However, there are several explanations for our results. First, rapamycin-treated animals underwent graft rejection, therefore their low Treg frequency could be associated to the allogenic response rather than hindering of Treg expansion. Second, rapamycin downregulates the ability of monocytic myeloid-derived suppressor cells (Mo-MDSC) to stimulate Treg expansion, while tacrolimus does not affect Mo-MDSC function [44]. Third, the tacrolimus dose used in this study is low, and it has been proposed that the immunosuppressive dosage, rather than the drug itself, is the key factor to inhibiting/allowing Treg expansion [45]. Moreover, a recent clinical study showed that tacrolimus-minimizing protocols enhance graft and patient survival [46]. Fourth, animals treated with tacrolimus exhibited a slower turnover of the graft T-cell compartment, which seems to be a necessary condition to tip the balance toward regulatory circuits. This hypothesis is based in reports from Megan Sykes group, showing that ITx patients exhibiting persistent blood macrochimerism, and consequently a lower rejection rate, also display slower influx of recipient T-cells into the graft [32].

Finally, we examined the correlation between heightened Treg frequencies and ACR prevention by employing an experimental group where tacrolimus treatment was withdrawn after CD4^+^CD25^high^FoxP3^+^ cell expansion (day 7 after surgery). Similar to the Leuven Immunomodulatory Protocol [14], an augmented Treg frequency was associated with a lower rejection rate, and recipients remained free of ACR for 1 week after discontinuation of the immunosuppressive treatment, a timeframe proved to be sufficient for developing tissue damage in untreated animals. Furthermore, at the endpoint, animals still exhibited T-cell blood chimerism, CD4 and CD8 frequencies similar to controls, and none of the pro-inflammatory genes assessed in graft samples were up-regulated. Principal component analysis confirmed these findings; with all samples obtained from this last experimental group clustering together and separately from those with ACR. Finally, IL-10, an anti-inflammatory mediator secreted by Tregs [5] and capable of modulating both intestinal damage in T-cell mediates enteropathy [47], and Th17 pathogenicity [48], was significantly augmented in serum samples after one week of discontinuing tacrolimus treatment. Of note, IL-17/TNF double positive T-lymphocytes have been described as key players in the intestinal allograft rejection [49]. Therefore, IL-10 inhibition of the allogenic response could be the underlying mechanism linking heightened Treg levels and graft protection. Nonetheless, alternative pathways not assessed in this study, such as immune-regulation through the PD-L1/PD-1 or CTLA-4/CD80-CD86 axis [5], among others [50], cannot be rule out. Importantly, the Treg frequency in both blood and graft remained above normal levels at the endpoint, suggesting that Tregs could still protect against ACR. Despite these positive results, we acknowledge that they cannot be considered as effective tolerance, but only as new evidence reinforcing the importance of regulatory circuits in solid organ transplantation.

In summary, our clinical and experimental findings highlight the link between Treg frequency and ACR, suggesting a crucial role for this lymphocyte subset in the protection of intestinal grafts. Consequently, pharmacological intervention targeting Treg expansion and Treg adoptive cell therapies hold potential for mitigating the rejection rate, paving the way for the development of more efficient therapeutic strategies. In this context, experimental models will play a pivotal role in validating and refining these approaches before their clinical translation.

## Supporting information

S1 Fig **(A)** Treg count and type of conditioning immunosuppression; non T-cell depleting (NTCD): basiliximab or rituximab, T-cell depleting (TCD): thymoglobulin or alemtuzumab. **(B)** Treg count and number of mismatches (ABDR). **(C)** Treg count and DSA presence/absence. Grey dots: patients without graft rejection; Black dots: patients with graft rejection. NTCD: Non T-cell depleting therapy; TCD: T-cell depleting therapy; DSA: Donor specific antibodies.
(PDF)

S2 Fig **(A)** Rapamycin blood levels (ng/mL) at the endpoint (day 10‒14 after surgery). **(B)** Representative dot plots of the gating strategy used to characterize donor/recipient CD4 and CD8 T lymphocytes and regulatory T-cells (CD4^+^CD25^high^FoxP3^+^). Singlets were excluded by FSC-A and FSC-H parameters. **(C)** Tacrolimus blood levels (ng/mL) at days 3, 7 and 9 after surgery. Dotted line indicates the technique detection limit. Animals received immunosuppression for 1 week after transplant and were sacrificed at day 14.
(PDF)

S1 Table Immunization status. (PDF)

S1 File (DOCX)

## Abbreviations

ACR: acute cellular rejection
MHC: major histocompatibility complex
CCL-11: eosinophil chemotactic protein
CXCL-10: interferon gamma-induce protein 10
IDO: indoleamine 2,3-dioxygenase
IFNγ: interferon gamma
IL-6: interleukin 6
IL-10: interleukin 10
IL-13: interleukin 13
IL-22: interleukin 22
ITx: intestinal transplantation
MCP-1: monocyte chemoattractant protein one
Mo-MDSC: monocytic myeloid-derived-suppressor-cell
TGFβ: transforming growth factor beta
Treg: regulatory T cell
FoxP3: forkhead box protein P3
Rapa: rapamycin
Tac: tacrolimus
