# Supplementary figures and images for: Low regulatory T-cells frequency is associated with graft rejection after small bowel transplantation: Clinical and experimental evidence
Source: PLoS One. 2025 Jan 24;20(1):e0307534. doi: 10.1371/journal.pone.0307534 (PMC11761612; doi:10.1371/journal.pone.0307534)

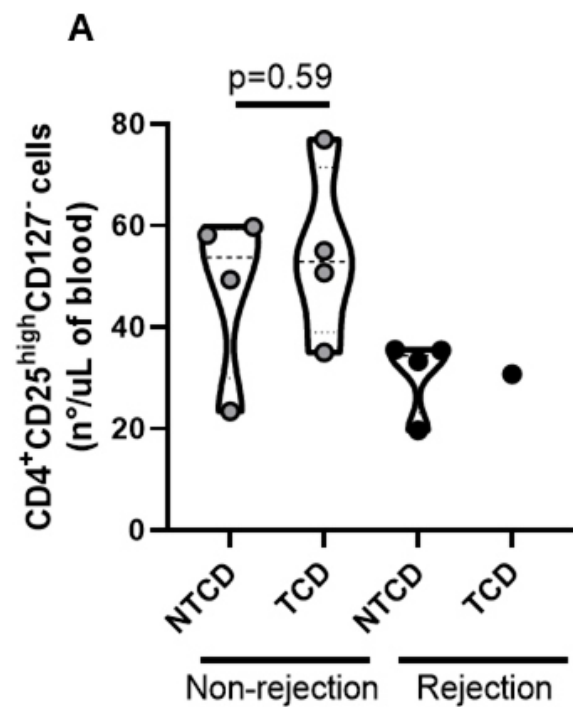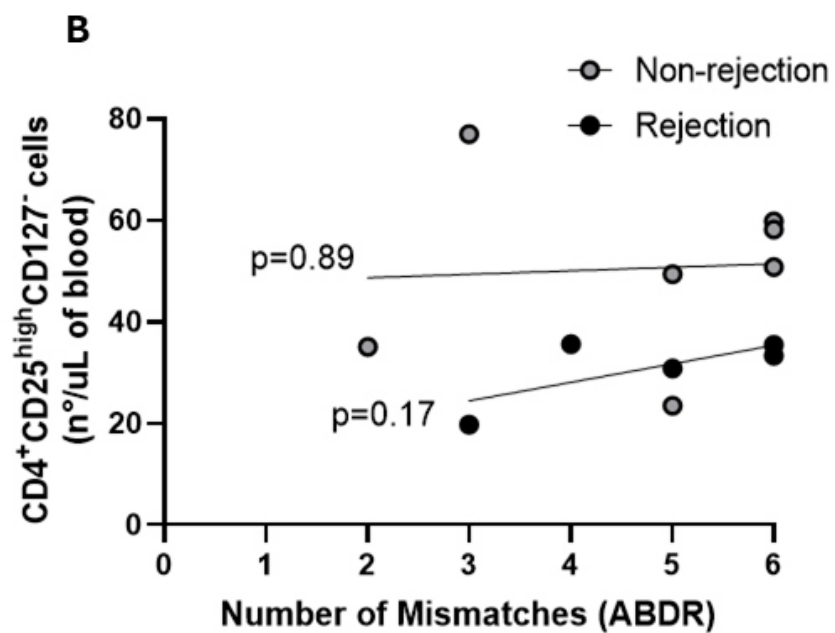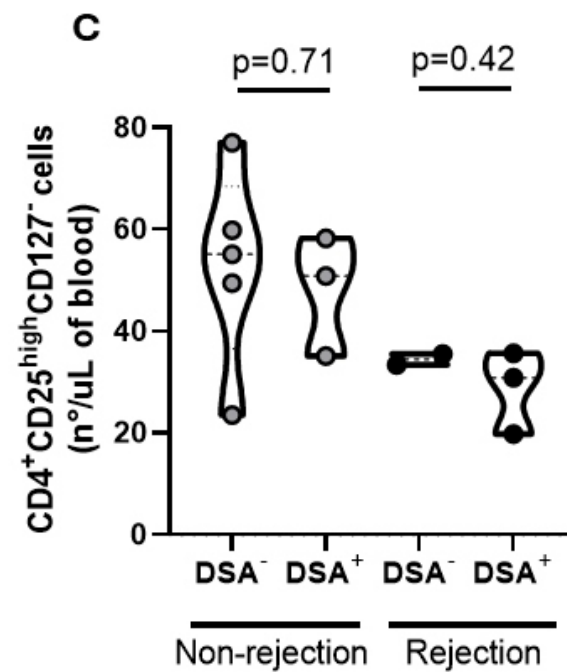

Supplement: S1 Fig — (A) Treg count and type of conditioning immunosuppression; non T-cell depleting (NTCD): basiliximab or rituximab, T-cell depleting (TCD): thymoglobulin or alemtuzumab. (B) Treg count and number of mismatches (ABDR). (C) Treg count and DSA presence/absence. Grey dots: patients without graft rejection; Black dots: patients with graft rejection. NTCD: Non T-cell depleting therapy; TCD: T-cell depleting therapy; DSA: Donor specific antibodies. (PDF) [file pone.0307534.s001.pdf]

**A**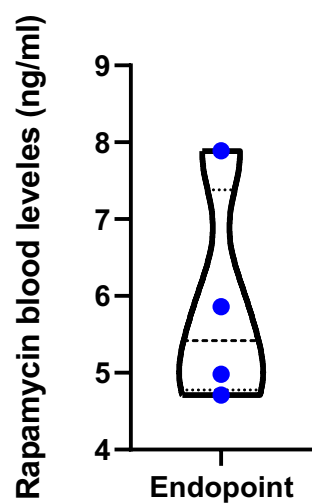**B**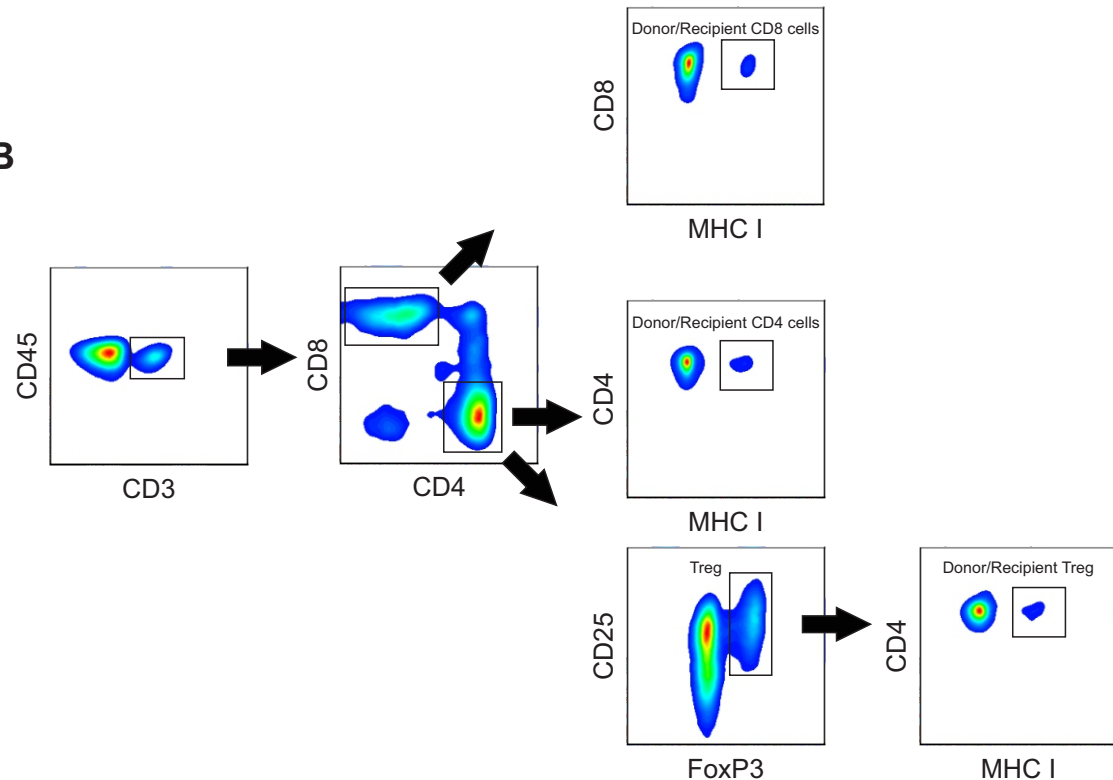**C**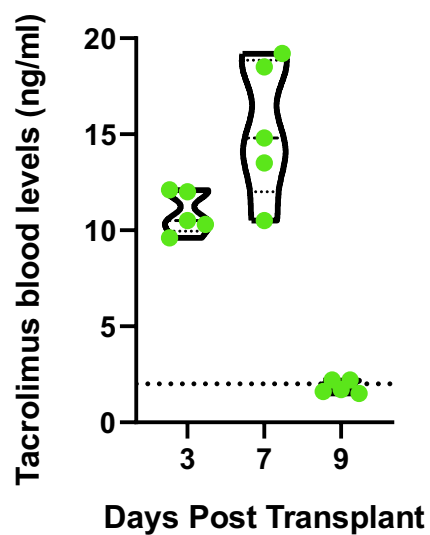

Supplement: S2 Fig — (A) Rapamycin blood levels (ng/mL) at the endpoint (day 10‒14 after surgery). (B) Representative dot plots of the gating strategy used to characterize donor/recipient CD4 and CD8 T lymphocytes and regulatory T-cells (CD4+CD25highFoxP3+). Singlets were excluded by FSC-A and FSC-H parameters. (C) Tacrolimus blood levels (ng/mL) at days 3, 7 and 9 after surgery. Dotted line indicates the technique detection limit. Animals received immunosuppression for 1 week after transplant and were sacrificed at day 14. (PDF) [file pone.0307534.s002.pdf]
