# Supplementary material for: Low regulatory T-cells frequency is associated with graft rejection after small bowel transplantation: Clinical and experimental evidence
Source: PLoS One. 2025 Jan 24;20(1):e0307534. doi: 10.1371/journal.pone.0307534 (PMC11761612; doi:10.1371/journal.pone.0307534)
Supplement: S1 Table — (PDF) [file pone.0307534.s003.pdf]

Table S1: Immunization Status

Pt#	Immunization status before TX
**1**	No available data from the patient childhood. HBV-; HAV-; VIH -; VH6 -; CMV IgG+ and IgM-; EBV -
**2**	CMV IgG -, VIH -.
**3**	HCV -; VIH -; HBV -;CMV -;VEB IgG +, IgM -
**4**	HSV -;CMV -; VEB -
**5**	CMV-; EBV -
**6**	CMV -; EBV -
**7**	CMV -; EBV -
**8**	CMV -; EBV -
**9**	CMV -; EBV -; Norovirus +
**10**	CMV-; EBV IgG+, IgM -
**11**	ACHBs +;HgHbs -;AcHBC -;HCV -;CMV-;EMV IgM +;
**12**	CMV IgG -; PEVENAR; MENVEO (meningococco quadrivalent ACW135Y+); HAV -; Bxero
**13**	CMV +; EBV -
**14**	HAV IgG +; HBV -; bcore:-;Bs Ag:-; HCV; HIV; Chagas -; CMV -; EBV -; Toxo -; Chickenpox IgG +; Rubella:-; $HTLV_Q$ -; HSV_1 -; HSV_2 -
**15**	Brucellosis -; Chagas -; HBsAg-; HCV -; HIV -; HTLV -;Syphilis -; BCG; DPT; IPV;HiB; MMR; PNEUMO; Anti-Flu vaccine; Meningococcus B-C; a-c; HBV.E
**16**	Brucellosis -; Chagas -; HBsAg-; HCV -; HIV -; HTLV -;Syphilis -; BCG; DPT; IPV;HiB; MMR; PNEUMO; Anti-Flu vaccine; Meningococcus B-C; a-c; HBV.
**16**	Brucellosis -; Chagas -; HBsAg-; HCV -; HIV -; HTLV -;Syphilis -; BCG; DPT; IPV;HiB; MMR; PNEUMO; Anti-Flue vaccine; Meningococcus B-C; a-c; HBV.
**17**	BCG, quintuple, 2 dosi HAV, MMR y Chickenpox. VDRL -, HIV -, HCV -, Anti HBS +, Measles IgG +, rubella IgG +, CMV IgG -, EBV IgG -, Toxo -, Chickenpox IgG +
**18**	CMV +; Brucellosis -; Chagas -; HBV -; HCV -; HIV -; HTLV -; Syphilis -; BCG; dpt; OPV; MEN BC; PRENUMO; SINAGYS
**19**	CMV +; Brucellosis -; Chagas -; HBV -; HCV -: HIV -; HTLV -; Syphilis -; BCG; dpt; OPV; MEN BC; PRENUMO; SINAGYS
**20**	CMV +; EBV +; HAV IgG+; HBV -; HCV-; HIV:-; Chagas-
**21**	Brucellosis -; chagas -; HBV -; HCV -; HIV -; HTLV -; Syphilis -; BCG;DPT;OPV;MEN BC; PNEUMO; SYNAGYS
**22**	Brucellosis -; Chagas:-; HBV -; HCV -; HIV -; HTLV -; Syphilis -; BCG; DPT; OPV; Hib: SAR: MMR
**23**	Brucellosis -; Chagas -; HAV IgG+; HBV -; HCV -; HIV -; HTLV -; Syphilis -; Toxo -; Chickenpox IgG-; Rubella +; BCG; DPT; HiB; HAV; HBV; MMR;OPV; PNEUMO; Anti-flu vaccine;

HAV: Hepatitis A
HBV: Hepatitis B
HCV: Hepatitis C
HSV: Herpes simplex Virus
CMV: Cytomegalovirus

EBV: Epstein-Barr Virus
VH6: Human herpes virus 6
HTLV: Human T-lymphotropic Virus
HIV: Human Immunodeficiency Virus
Toxo: Toxoplasmosis

BCG: Bacille Calmette-Guérin
DPT: Diphtheria, Tetanus and Pertussis
HiB: Hemophilus influenzae type b
IPV: Inactivated polio vaccine
MMR: Measles, mumps, and rubella
