# Supplementary material for: Low regulatory T-cells frequency is associated with graft rejection after small bowel transplantation: Clinical and experimental evidence
Source: PLoS One. 2025 Jan 24;20(1):e0307534. doi: 10.1371/journal.pone.0307534 (PMC11761612; doi:10.1371/journal.pone.0307534)
Supplement: S1 File — (DOCX) [file pone.0307534.s004.docx]

**Low regulatory T-cells frequency is associated with graft rejection after small bowel transplantation: clinical and experimental evidence**

**SUPPLEMENTAL DIGITAL CONTENT**

**Study enrollment criteria:**

All patients under 18 years old who attended medical appointments for routine control, or for symptoms of rejection, and had Treg determination performed between 2018-2020. For those included in the rejection group, the diagnoses had to be confirmed by endoscopic and histological findings. Patients enrolled in the non-rejection group showed no clinical signs compatible with rejection at least 3 months before sample collection.

**Isolation of lamina propria cells (detailed protocol):**

To remove cells and mucus, the tissue (3 cm) was chopped into 1 mm pieces and placed in Hank’s Balanced Salt Solution medium without Ca^2+^ and Mg^2+^ (Gibco, Grand Island, NY, USA), supplemented with 10% fetal bovine serum, antibiotics, 0.5 mM ethylenediminetetraacetic acid, and 0.1 mM dithiothreitol (for 30 min at 37°C). The supernatant was discarded, and the remaining tissue was digested for 30 min at 37°C in Roswell Park Memorial Institute medium supplemented with collagenase type I (10 U/mL; Roche, Basel, Switzerland) and 10% fetal bovine serum. The cell suspension was filtered through a 40 µm nylon mesh, washed with sterile PBS, and suspended in PBS for flow cytometry assays.

**RNA isolation and real-time RT-PCR**

Total RNA from small bowel segments was extracted using Ilustra RNAspin Mini RNA Isolation Kit (GE Healthcare, Little Chalfont, UK) following the manufacturer’s protocol. The isolated RNA was reverse-transcribed using random primers and MML-V reverse transcriptase (Invitrogen, Carlsbad, CA, USA). Real-time quantitative PCR was performed using SYBR Green PCR Master Mix (Bio-Rad, Hercules, CA, USA) and an iCycler thermal cycler (Bio-Rad). The primer sequences are shown in Table 3.
